# Growth Mindset and Self-Perceived Adaptive Intelligence: A Structural Model of Motivation, Metacognition, Self-Regulated Learning, and Academic Adaptation

**DOI:** 10.3390/jintelligence14070133

**Published:** 2026-07-02

**Authors:** Aljawharah Fahad Aljubilah, Khaled Ahmed Abdel-Al Ibrahim, Ahmad Al-Adwan, Sayed M. Ismail, Anwar Hammad Al-Rashidi, Khalid Abdullah Alotaibi

**Affiliations:** 1 Department of Psychology, College of Education and Human Development, Princess Nourah bint Abdulrahman University, P.O. Box 84428, Riyadh 11671, Saudi Arabia; afaljbelh@pnu.edu.sa; 2 Department of Psychology, College of Education, Prince Sattam Bin Abdulaziz University, P.O. Box 173, Al-Kharj 11942, Saudi Arabia; a.alrashidi@psau.edu.sa (A.H.A.-R.); ka.alotaibi@psau.edu.sa (K.A.A.); 3 Business Technology Department, Al-Ahliyya Amman University, Amman 19328, Jordan; a.adwan@ammanu.edu.jo; 4 English Department, College of Sciences and Humanities, Prince Sattam Bin Abdulaziz University, P.O. Box 173, Al-Kharj 11942, Saudi Arabia

**Keywords:** self-perceived adaptive intelligence, adaptive intelligence theory, academic growth mindset, learning motivation, metacognitive awareness, self-regulated learning strategies, academic adaptation, learner autonomy, higher education, Jordan

## Abstract

Drawing on mindset theory, self-regulated learning theory, learner autonomy, and adaptive intelligence theory, this study tested an integrated structural model of the associations between academic growth mindset and self-perceived adaptive intelligence among Jordanian undergraduates. In this study, self-perceived adaptive intelligence refers to students’ perceived wisdom-related, social/practical, creative, and uncertainty-navigation tendencies; it is not an objective or performance-based measure of cognitive ability. The hypothesized sequential mediation structure was retained, but it was estimated and interpreted as a set of theoretically ordered indirect associations through learning motivation, metacognitive awareness, self-regulated learning strategies, and academic adaptation. Learner autonomy was examined as a moderator of the association between self-regulated learning strategies and academic adaptation. Data were obtained from 640 undergraduate students enrolled in Jordanian universities and analyzed using partial least squares structural equation modeling with WarpPLS 8.0. Academic growth mindset was positively associated with learning motivation and metacognitive awareness. Both constructs were positively associated with self-regulated learning strategies, which, in turn, were positively associated with academic adaptation; academic adaptation, in turn, was positively associated with self-perceived adaptive intelligence. The theoretically ordered sequential indirect associations through the motivational and metacognitive routes were statistically significant, whereas learner autonomy did not significantly moderate the association between self-regulated learning strategies and academic adaptation. Because the data were single-wave and self-reported, the term “sequential” refers to the theory-imposed ordering of paths in the statistical model, not to an observed temporal or developmental progression. Accordingly, the findings represent structural associations and do not establish causal sequencing. The findings contribute to intelligence and higher-education research by distinguishing domain-specific academic adaptation from broader self-perceived adaptive intelligence informed by Sternberg’s adaptive intelligence framework.

## 1. Introduction

The higher education sector is undergoing a major transformation as scholars increasingly recognize that academic achievement is not determined solely by students’ cognitive ability, prior achievement, or exposure to instructional content (Stankov & Lee, 2025). Successful learning is increasingly understood as involving interrelated psychological and behavioral processes through which students form beliefs about their capacities, mobilize motivational resources, monitor their cognitive functioning, regulate their learning strategies, and adjust to complex academic environments (Tinajero et al., 2024). Accordingly, recent research has moved beyond narrow achievement-based explanations toward more holistic models that examine how learners’ beliefs, motivation, metacognition, self-regulation, autonomy, adaptation, and intelligence-related self-perceptions are jointly associated with academic functioning (Gan et al., 2026; K. M. Xu et al., 2025; Lobos et al., 2024).

Growth mindset has emerged as a particularly influential construct, referring to the belief that ability is malleable and can be developed through sustained effort, persistence, feedback, and effective strategies (Kapasi & Pei, 2022; Younger et al., 2024). This belief is especially significant because students frequently encounter academic pressure, challenges, assessment anxiety, and obstacles to repeated performance. Thus, students who endorse a growth mindset are more likely to perceive difficulty as an opportunity for development rather than as evidence of fixed incapacity. While empirical studies substantiate the positive relationship between growth mindset and academic outcomes, a critical evaluation of the literature reveals important complexities. For example, Teng et al. (2024) found that the growth mindset was positively associated with self-regulated vocabulary learning and vocabulary knowledge among university students. Further, Bai et al. (2025) demonstrated that growth-oriented language mindset profiles correlated with greater use of self-regulated learning strategies and higher English achievement. Similarly, growth mindset indirectly enhanced integrated writing performance through autonomous motivation and strategy use, suggesting that the relationship between mindset and achievement may be mediated by other psychological variables (Zhang et al., 2024). Thus, while mindset serves as a motivational and strategic driver, its influence on learning behavior is complex and may depend on interactions between individual and contextual factors.

Growth mindset is grounded in implicit theories of intelligence, which encompass competing views regarding the nature of ability: entity theory posits that intelligence is fixed and unchangeable, whereas incremental theory holds that ability can be developed through effort and effective strategies (Dweck, 2006). These theoretical perspectives shape how students interpret effort, failure, and academic challenges. In education, where progress is incremental and mistakes are common, these beliefs become particularly salient. Learners who adopt an entity or fixed mindset may avoid challenging tasks, perceive errors as evidence of low innate ability, and disengage when faced with difficulty (Kapasi & Pei, 2022). In contrast, students who endorse a growth or incremental mindset are more likely to attribute progress to controllable factors such as effort, strategic learning, feedback use, and persistence. Synthesizing these perspectives, growth mindset is treated here as a distal psychological belief that may be associated with a theoretically ordered pattern of motivational, cognitive, regulatory, and adaptive variables rather than as evidence of a causal cascade.

When students believe that their abilities are improvable, they are more likely to value effort, sustain engagement, and set long-term learning goals (Jallali et al., 2026; Younger et al., 2024). Learning motivation may therefore help account for the association between growth mindset and self-regulated learning strategies by reflecting the psychological energy and persistence required for demanding academic work. This is particularly relevant in educational contexts, where progress depends on repeated practice and resilience in the face of errors. Bai and Wang (2023) found that growth mindset, self-efficacy, and intrinsic value jointly contributed to self-regulated learning and English achievement, while Zhang et al. (2024) identified an indirect association between growth mindset and writing performance through autonomous motivation and strategy use. Additional evidence indicates that academic growth mindset is associated with learning engagement (Pan et al., 2026) and that self-regulation interventions may yield motivational benefits (Sansone et al., 2026). Thus, learning motivation is positioned as a theoretically relevant intervening variable that links growth-oriented beliefs to purposeful learning behaviors (Lu et al., 2026).

Metacognitively aware students can identify knowledge gaps, select appropriate strategies, monitor progress, and adjust learning behaviors in response to feedback (Apperson et al., 2025). Recent research indicates that metacognition is closely associated with self-regulated learning and academic transitions, as students coordinate executive functions, learning strategies, and reflective monitoring during complex academic tasks (Dörrenbächer-Ulrich et al., 2024; Tuononen et al., 2023). Growth mindset may be positively associated with metacognitive awareness because students who believe learning can improve are more likely to perceive their cognitive processes as controllable and modifiable. Supporting this view, Prihandoko et al. (2024) found that metacognition statistically mediated the association between growth mindset and academic writing performance among undergraduates.

Self-regulated learning strategies are important because students are increasingly required to manage their learning independently, particularly in blended, digital, and student-centered contexts. Self-regulated learning reflects active control over learning processes rather than passive knowledge reception. Chou and Zou (2020) demonstrated that feedback mechanisms and learning tools support self-regulated learning activities, while Wirth et al. (2020) conceptualized self-regulated learning as a layered process involving cognitive, metacognitive, and motivational regulation. Recent studies also emphasize associations between self-regulated learning, academic transition, performance, and student well-being (Lobos et al., 2024; Verdugo & Martínez-Líbano, 2026). In the present model, self-regulated strategies are treated as behavioral correlates of motivational and metacognitive resources. Evidence from Arab university contexts further indicates that problematic technology use and self-regulation failures are associated with mind-wandering, cognitive failures, and lower academic life satisfaction (Al-Abyadh et al., 2024).

Self-regulated learners may report stronger academic adaptation because they possess strategic tools for organizing learning, monitoring progress, seeking support, and modifying behavior in response to academic obstacles. Academic adaptation encompasses students’ adjustment to university requirements, learning demands, assessment systems, institutional expectations, and changing academic circumstances (Shamionov et al., 2020). Chen (2025), for example, identified learning adaptation and online self-regulated learning as intervening variables in the association between digital literacy and academic achievement among college students. Academic adaptation is therefore treated in this study as a domain-specific correlate of self-regulated learning rather than as a demonstrated developmental outcome.

Autonomous learners may be better positioned to apply self-regulated learning strategies across changing academic demands because autonomy reflects learner agency, responsibility, motivation, regulation, and flexible strategy use (Gupta et al., 2024). In contrast, students with lower autonomy may report using certain learning strategies while remaining reliant on external guidance. Gupta et al. (2024) conceptualized learner agency as a critical perspective for understanding self-regulated and self-directed learning, while Liu et al. (2026) emphasized the multidimensional nature of English-language learner autonomy assessment. Namaziandost et al. (2024) also found that learner autonomy and academic engagement were positively associated with EFL students’ willingness to communicate, foreign-language learning self-esteem, and L2 grit. These findings support examining learner autonomy as a possible moderator of the association between self-regulated learning strategies and academic adaptation.

Adaptive intelligence theory broadens traditional conceptions of intelligence by emphasizing effective responses to current challenges, anticipation of future problems, flexible application of knowledge, and constructive judgment in dynamic real-world contexts (Sternberg, 2019, 2021). The present study does not assess adaptive intelligence as an objectively measured cognitive ability. Instead, it operationalizes Self-perceived adaptive intelligence as students’ perceived tendencies toward wisdom-related judgment, social/practical reasoning, creative problem solving, and navigation of uncertainty. This attitudinal and self-perceptual operationalization is consistent with Sternberg et al.’s (2024) emphasis on the adaptively intelligent attitude, but it should not be interpreted as equivalent to performance on an ability test. Growth mindset, self-regulation, and academic adaptation are therefore examined as correlates of these self-perceived adaptive tendencies rather than as causes of a measured intellectual capacity.

Despite growing research on growth mindset, motivation, metacognition, self-regulated learning, autonomy, and academic adjustment, important gaps remain. Existing studies are conceptually fragmented and rarely integrate these constructs within a unified structural model (Dörrenbächer-Ulrich et al., 2024; Theobald et al., 2026). First, growth mindset has often been examined as a direct predictor of learning outcomes, with limited attention to the intervening variables that may statistically account for its association with strategic learning behaviors (K. M. Xu et al., 2025; Teng et al., 2024; Zhang et al., 2024). Second, although learning motivation and metacognitive awareness have each been linked to self-regulated learning, few studies have examined their combined explanatory role (Dörrenbächer-Ulrich et al., 2024; Henry & Liu, 2024; K. M. Xu et al., 2025; Tinajero et al., 2024). Third, prior research has largely focused on discrete academic outcomes, whereas academic adaptation and broader self-perceived adaptive intelligence remain less examined together (Bai et al., 2025; Sternberg, 2021; Teng et al., 2024; Verdugo & Martínez-Líbano, 2026; Zhang et al., 2024). Fourth, the moderating role of learner autonomy in the association between self-regulated learning strategies and academic adaptation remains insufficiently explored (Namaziandost et al., 2024; Núñez-Regueiro et al., 2025; Radović et al., 2024). Finally, evidence remains limited in Jordanian higher education, where academic, institutional, and sociocultural conditions may be relevant to the observed pattern of associations.

This study addresses these gaps by testing an integrated structural model among undergraduate students in Jordanian universities. Specifically, it evaluates two theoretically ordered sequential indirect pathways linking academic growth mindset with self-perceived adaptive intelligence: one involving learning motivation, self-regulated learning strategies, and academic adaptation, and the other involving metacognitive awareness, self-regulated learning strategies, and academic adaptation. The study also examines whether learner autonomy moderates the association between self-regulated learning strategies and academic adaptation. The sequential ordering is retained because it follows the proposed theoretical framework; however, the estimated paths are interpreted as statistical decompositions of cross-sectional associations rather than evidence that the variables unfolded in that temporal order.

## 2. Theoretical Underpinnings and Hypotheses Development

### 2.1. Theoretical Underpinnings

This study is grounded in an integrated framework connecting mindset theory, self-regulated learning theory, learner autonomy, and adaptive intelligence theory. The framework specifies theoretically directional associations among a foundational belief about ability, motivational and metacognitive resources, reported learning strategies, academic adaptation, and Self-perceived adaptive intelligence (Dweck, 2006; K. M. Xu et al., 2025). Mindset theory suggests that students who believe ability can be developed tend to interpret academic difficulty as more manageable, sustain effort, seek feedback, and revise ineffective strategies. Self-regulated learning theory further suggests that motivation and metacognitive awareness are associated with the reported use of strategic learning behaviors (Efklides, 2011; Panadero, 2017; Zimmerman, 2000). Accordingly, learning motivation and metacognitive awareness are modeled as statistical intervening variables in the associations between academic growth mindset and self-regulated learning strategies.

Adaptive intelligence theory emphasizes effective responses to current challenges, anticipation of future problems, and flexible application of knowledge under uncertainty (Sternberg, 2021). In this study, the outcome represents Self-perceived adaptive intelligence rather than measured intellectual ability. Academic adaptation is expected to be positively associated with this broader self-perceived functioning because students who report successful adjustment to academic demands may also report greater flexibility, contextual awareness, strategic revision, and problem-solving tendencies. Learner autonomy is also examined as a possible moderator, with students with greater perceived agency applying self-regulated strategies more flexibly across changing academic situations (Gupta et al., 2024; Lou & Zarrinabadi, 2022).

**Figure 1:**
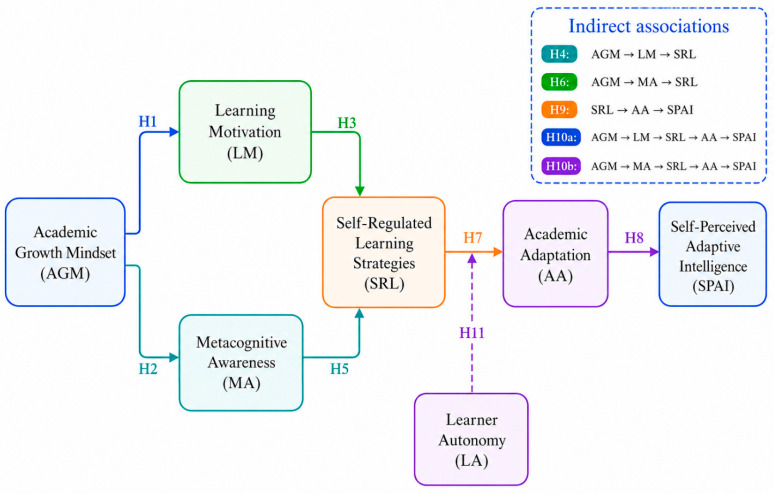
Proposed research framework. Source: Author’s preparation.

Overall, the framework shown in Figure 1 retains the hypothesized sequential mediation structure while representing it empirically as a set of theoretically ordered direct and indirect associations. In the present single-wave design, the sequence is imposed by theory rather than observed over time. The model, therefore, does not establish that a growth mindset temporally precedes the intervening variables or that academic adaptation produces subsequent changes in self-perceived adaptive intelligence.

### 2.2. Conceptual Development of the Hypotheses

#### 2.2.1. Academic Growth Mindset and Learning Motivation

Academic growth mindset refers to students’ belief that academic ability can be developed through effort, effective strategies, feedback, and persistence rather than being determined by fixed talent (Dweck, 1999; Mercer & Ryan, 2010; S. Ryan & Mercer, 2012). This belief shapes how students interpret difficulty, failure, and feedback. Students with a stronger growth mindset are more likely to view challenges as opportunities for improvement, whereas students with a fixed mindset may perceive difficulty as evidence of limited ability and disengage from demanding tasks (Lou & Noels, 2016).

The association between academic growth mindset and learning motivation is grounded in achievement motivation theory, which links ability beliefs with students’ goals, effort investment, and responses to success or failure (Dweck, 1999). Growth-minded students tend to adopt mastery goals, value effort, seek feedback, and sustain engagement because they perceive improvement as attainable (Blackwell et al., 2007; Lou & Noels, 2017). This logic is also consistent with self-determination theory, which associates motivation with experiences of competence, autonomy, and purposeful engagement. Bai and Wang (2023) found that growth mindset, self-efficacy, and intrinsic value jointly predicted self-regulated learning and English achievement, while Zhang et al. (2024) reported an indirect association between growth mindset and writing performance through autonomous motivation and strategy use. Accordingly, students with stronger academic growth-mindset beliefs are expected to report higher learning motivation.

**H1.**  *Academic growth mindset is positively associated with students’ learning motivation.*

#### 2.2.2. Academic Growth Mindset and Metacognitive Awareness

Metacognitive awareness refers to students’ knowledge of their own cognitive processes and their capacity to plan, monitor, regulate, and evaluate learning activities (Efklides, 2011; Panadero, 2017). It consists of two interrelated dimensions: metacognitive knowledge, which concerns students’ understanding of how learning occurs and which strategies are effective, and metacognitive regulation, which involves the active control of learning through planning, monitoring, and evaluation. Thus, metacognitive awareness is central to academic development because it enables students to identify learning difficulties, select appropriate strategies, evaluate progress, and adjust ineffective approaches in response to changing academic demands (Dörrenbächer-Ulrich et al., 2024; K. M. Xu et al., 2025).

On the other hand, the academic growth mindset provides a strong theoretical basis for metacognitive awareness. Students who believe that academic ability can be developed are more likely to view learning as controllable, improvable, and responsive to strategic effort. This belief encourages learners to reflect on how they learn, evaluate the effectiveness of their strategies, and revise their approaches when difficulties arise (K. M. Xu et al., 2025). By contrast, students with fixed-ability beliefs may perceive academic performance as predetermined, which can reduce the perceived value of monitoring, feedback use, and strategy adjustment. Recent research supports this argument, showing that the growth mindset is closely associated with self-regulated learning processes, including planning, monitoring, strategy use, and adaptive learning behavior (K. M. Xu et al., 2025; Prihandoko et al., 2024).

A growth mindset is empirically linked to metacognitive functioning. For instance, Prihandoko et al. (2024) found that metacognition mediated the relationship between growth mindset and academic writing performance, suggesting that students’ mindset beliefs shape how they monitor and regulate learning. Similarly, W. Xu et al. (2025) demonstrated that growth mindset predicted metacognitive strategy use, which subsequently contributed to learning performance. Recent reviews also emphasize that metacognitive strategies are essential to self-regulated learning because they allow students to plan, monitor, and evaluate their academic activities (Dörrenbächer-Ulrich et al., 2024; K. M. Xu et al., 2025). Therefore, students with stronger academic growth mindsets are expected to demonstrate greater metacognitive awareness because they perceive learning improvement as achievable through reflection, feedback, and deliberate strategy regulation. Accordingly,

**H2.**  *Academic growth mindset is positively associated with students’ metacognitive awareness.*

#### 2.2.3. Academic Growth Mindset, Learning Motivation, and Self-Regulated Learning Strategies

Self-regulated learning strategies refer to the deliberate cognitive, metacognitive, motivational, and behavioral actions through which students actively manage their academic learning. These strategies include goal setting, planning, attention control, effort regulation, resource management, self-monitoring, feedback seeking, and self-evaluation (Oxford, 2017; Zimmerman, 2000). Such strategies are particularly important in higher education, where students are increasingly expected to regulate their learning independently within digital, blended, and student-centered learning environments (Lobos et al., 2024).

Learning motivation represents a central antecedent of self-regulated learning because strategic regulation requires sustained effort, persistence, and purposeful engagement (Bakhtiar & Hadwin, 2022). Motivated students are more likely to establish academic goals, allocate cognitive and temporal resources, monitor their learning progress, seek feedback, and modify their strategies when learning outcomes are unsatisfactory (Hemmler & Ifenthaler, 2024; Henry & Liu, 2024). By contrast, students with weak motivation may understand the value of self-regulated learning strategies but fail to apply them consistently, particularly when academic tasks are demanding or learning outcomes are delayed (Bai & Wang, 2023; Zhang et al., 2024). Therefore, motivation provides the psychological energy that enables students to translate learning intentions into systematic regulatory action. Accordingly, the following hypothesis is proposed:

**H3.**  *Learning motivation is positively associated with students’ self-regulated learning strategies.*

Learning motivation may also statistically account for part of the association between academic growth mindset and self-regulated learning strategies. Students who believe that academic ability can be developed are more likely to interpret effort as meaningful, setbacks as manageable, and improvement as attainable (Dweck & Yeager, 2019; K. M. Xu et al., 2025). These beliefs are associated with motivational readiness and a willingness to engage in planning, monitoring, feedback-seeking, effort regulation, and strategy revision. This argument is consistent with self-regulated learning theory, which identifies motivation as an important correlate of the initiation and maintenance of strategic learning behaviors (Bakhtiar & Hadwin, 2022; Zimmerman & Schunk, 2008). Accordingly, the following indirect-association hypothesis is proposed:

**H4.**  *Academic growth mindset has a positive indirect association with students’ self-regulated learning strategies through learning motivation.*

#### 2.2.4. Academic Growth Mindset, Metacognitive Awareness, and Self-Regulated Learning Strategies

Metacognitive awareness serves as a foundational antecedent to self-regulated learning, enabling students to interpret task demands, monitor their understanding, evaluate the effectiveness of learning strategies, and adjust their behavior when progress is insufficient (Efklides, 2011; Panadero, 2017). According to Zimmerman’s cyclical model, effective self-regulation requires forethought, performance monitoring, and self-reflection. Each phase relies on students’ ability to evaluate their knowledge, learning processes, and the appropriateness of their strategies for the given task. Recent systematic evidence demonstrates that metacognition is closely linked to self-regulated learning, especially during academic transitions, when students are expected to take greater responsibility for planning, monitoring, and adapting their learning (Dörrenbächer-Ulrich et al., 2024).

Although students may recognize the importance of planning, monitoring, and evaluation, they often fail to implement these strategies when motivation is low, feedback is insufficient, or the learning environment does not facilitate strategic regulation (Cervin-Ellqvist et al., 2021; Zimmerman & Moylan, 2009). Recent research emphasizes that self-regulated learning is not solely a cognitive or metacognitive process; it is also a context-sensitive, technology-mediated behavior influenced by instructional design, digital tools, and learner engagement. Reviews of self-regulated learning in digital and blended contexts indicate that effective regulation requires comprehensive support throughout the learning cycle, rather than mere awareness of strategies (Faza & Lestari, 2025; Prasse et al., 2024). This is particularly important in digitally saturated environments, where smartphone addiction and self-regulation failures have been shown to intensify mind wandering and cognitive failures and reduce academic life satisfaction among university students (Al-Abyadh et al., 2024).

Another critical issue involves the measurement of self-regulated learning. Much of the existing research relies on self-report instruments; however, students’ reported awareness of strategies may not accurately reflect their actual regulatory behaviors during learning. Studies employing learning analytics and trace data have demonstrated that behavioral data can capture self-regulated learning processes differently from self-reports and, in some cases, predict academic performance more effectively (Alhazbi et al., 2024; Ye & Pennisi, 2022). Nevertheless, trace-data approaches present limitations, as raw digital actions must be systematically mapped onto meaningful self-regulated learning constructs, and this methodological translation remains a significant challenge (Boulahmel et al., 2025; Osakwe et al., 2024).

Collectively, these perspectives indicate that metacognitive awareness is closely associated with effective self-regulation but is not sufficient on its own. Its primary value lies in students’ capacity to connect reflective understanding with planning, monitoring, and strategy revision. Consequently, students reporting greater metacognitive awareness are expected to report more frequent use of self-regulated learning strategies. For instructors, this literature underscores the value of learning environments that foster metacognitive skills and provide opportunities to practice self-regulation through guided reflection, scaffolded feedback, and explicit strategy instruction.

**H5.**  *Metacognitive awareness is positively associated with students’ self-regulated learning strategies.*

Metacognitive awareness is also proposed as an intervening variable in the association between academic growth mindset and self-regulated learning strategies. Self-regulation theory positions strategic learning at the intersection of motivational beliefs and metacognitive regulation (Efklides, 2011). Metacognitive awareness is associated with students’ capacity to plan, monitor, evaluate, and adjust cognitive effort during academic tasks (Panadero, 2017; Winne & Azevedo, 2022). Dörrenbächer-Ulrich et al. (2024) likewise identified metacognition as a central component of self-regulated learning, particularly during academic transitions.

Mindsets refer to students’ beliefs about the malleability of ability (Dweck, 2006; Dweck & Yeager, 2019). An academic growth mindset holds that academic competence can be developed through effort, persistence, feedback, and effective strategies. In the present model, academic growth mindset is expected to be positively associated with metacognitive awareness for several reasons. Students who view ability as malleable may place greater value on monitoring and revising their learning strategies. Such beliefs may also direct attention toward how learning occurs, which strategies are effective, and how progress can be improved. When difficulties arise, a growth mindset may be associated with fewer maladaptive attributions and greater willingness to continue monitoring and regulating learning. Recent evidence supports this reasoning by showing positive associations between growth mindset and self-regulated learning strategies and by emphasizing behavioral and regulatory mechanisms rather than assuming a simple direct effect (K. M. Xu et al., 2025; Mendoza & Yan, 2025).

Empirical research provides further support for this proposed indirect association. K. M. Xu et al. (2025) reported an overall positive association between growth mindset and self-regulated learning strategies and emphasized the need to examine intervening mechanisms. Mendoza and Yan (2025) similarly described growth-oriented practices as including proactive and reflective learning behaviors such as planning, monitoring, adapting strategies, and responding constructively to feedback. Accordingly, metacognitive awareness is modeled as a cognitive intervening variable through which academic growth mindset may be statistically associated with self-regulated learning strategies (Dörrenbächer-Ulrich et al., 2024; K. M. Xu et al., 2025; Winne & Azevedo, 2022).

**H6.**  *Academic growth mindset has a positive indirect association with students’ self-regulated learning strategies through metacognitive awareness.*

#### 2.2.5. Self-Regulated Learning Strategies, Academic Adaptation, and Self-Perceived Adaptive Intelligence

Academic adaptation refers to students’ capacity to adjust effectively to the cognitive, behavioral, and affective demands of university learning (Monteiro & Soares, 2023). It reflects students’ ability to revise ways of thinking, modify learning behaviors, seek academic and institutional resources, and regulate negative emotions when they encounter unfamiliar, changing, or uncertain academic situations (Han, 2024; Martin et al., 2012). In higher education, academic adaptation is particularly important because students are expected to cope with greater autonomy, complex assignments, diverse assessment practices, and reduced external monitoring. Recent studies have therefore treated academic adjustment as a central indicator of students’ academic functioning, persistence, and well-being during the university experience (Cao & Tang, 2025; Cobo-Rendón et al., 2023; Oliveira Silva et al., 2025).

From a self-regulation perspective, students with stronger self-regulation strategies are better able to organize their learning, set academic goals, monitor progress, manage effort, seek feedback, and modify ineffective approaches (Cazan, 2012; van Rooij et al., 2018). In this regard, self-regulated strategies provide students with the behavioral and metacognitive tools needed to respond productively to academic challenges. They enable students to move beyond passive adjustment by actively interpreting task demands, identifying barriers, and selecting appropriate strategies to meet changing university requirements (Cazan, 2012; van Rooij et al., 2018). Recent evidence also confirms that self-regulated learning is particularly important for college students because they are required to manage academic stress, independent study, competing responsibilities, and increasingly self-directed learning environments (Jan & Parveen, 2025).

In this study, self-regulated learning strategies are expected to be positively associated with academic adaptation. Students who report using planning and goal-setting strategies may be better able to structure academic tasks and respond to unfamiliar learning demands. Those who monitor progress and evaluate performance may identify difficulties earlier and revise their strategies, while students who regulate effort, manage time, seek support, and revise approaches may report stronger adjustment to complex assignments, changing assessment expectations, and emotional pressure (Alsetoohy et al., 2026). Prior evidence has shown associations between self-regulated learning strategies and academic adjustment (Cazan, 2012; Raković et al., 2022). Chen (2025) further found that learning adaptation and online self-regulated learning jointly accounted for variance in academic achievement. These findings support testing the following association:

**H7.**  *Self-regulated learning strategies are positively associated with academic adaptation.*

Academic adaptation refers here to domain-specific adjustment to the cognitive, behavioral, and affective demands of university learning. Its items explicitly ask whether students can revise their thinking, change task-related behavior, seek resources, and regulate emotions in new or uncertain academic situations. By contrast, self-perceived adaptive intelligence is a broader, theory-informed self-perception encompassing wisdom-related judgment, social/practical orientations, creative problem-solving, and the navigation of uncertainty across academic and everyday problems (Sternberg, 2019, 2021; Sternberg et al., 2024). Thus, the two constructs are theoretically related but not interchangeable: academic adaptation concerns adjustment within the university context, whereas self-perceived adaptive intelligence concerns broader perceived tendencies in problem solving and contextual judgment. This distinction was supported empirically by the cross-construct HTMT results: values between the three academic-adaptation dimensions and the four self-perceived adaptive-intelligence dimensions ranged from 0.085 to 0.505, with the highest value (0.505, between cognitive adaptation and wisdom-related adaptation) remaining well below the 0.85 criterion. The latter should not be interpreted as an objectively measured cognitive capacity.

The proposed association between the constructs, therefore, tests whether students who report stronger domain-specific academic adjustment also report broader adaptive tendencies. Prior research links cognitive flexibility, self-regulation, resilience, adaptability, and practical judgment, while treating these as distinguishable constructs (Bordbar et al., 2024; Karakuş, 2024; Nakhostin-Khayyat et al., 2024). On this basis, a positive association is expected, without assuming that academic adaptation causes or leads to self-perceived adaptive intelligence.

**H8.**  *Academic adaptation is positively associated with self-perceived adaptive intelligence.*

van Rooij et al. (2018) argued that academic adjustment statistically accounts for part of the association between self-regulated behavior and academic success. In the present model, academic adaptation is likewise examined as an intervening variable linking self-regulated learning strategies with broader self-perceived adaptive intelligence. This specification is consistent with research linking adaptability to the cognitive, behavioral, and emotional dimensions of self-regulated learning (Feraco et al., 2023) and with Sternberg et al.’s (2024) distinction between adaptive abilities and adaptive attitudes. The proposed indirect effect is interpreted as a cross-sectional statistical association rather than evidence that regulatory strategies temporally produce adaptive functioning.

**H9.**  *Self-regulated learning strategies have a positive indirect association with Self-perceived adaptive intelligence through academic adaptation.*

#### 2.2.6. Academic Growth Mindset and Theoretically Ordered Sequential Indirect Associations with Self-Perceived Adaptive Intelligence

The proposed model retains two theoretically ordered sequential indirect associations linking academic growth mindset to self-perceived adaptive intelligence. In the motivational pathway, students with stronger growth-mindset beliefs are expected to report greater learning motivation, which is associated with more frequent use of self-regulated learning strategies (Blackwell et al., 2007; Burnette et al., 2013; Panadero, 2017; R. M. Ryan & Deci, 2020; Yeager & Dweck, 2020). The sequence reflects the model’s theoretical specification and should not be read as evidence that these variables changed in this order over time.

In the metacognitive route, academic growth mindset is expected to be positively associated with metacognitive awareness, which is, in turn, associated with self-regulated learning strategies. This ordering is grounded in the literature that links growth mindset to metacognitive strategy use and defines metacognition as the awareness and regulation of learning processes (K. M. Xu et al., 2025; Panadero, 2017; W. Xu et al., 2025).

Self-regulated learning strategies are expected to be associated with academic adaptation because planning, monitoring, effort regulation, help-seeking, time management, and self-evaluation are relevant to adjustment to university demands (Cazan, 2012; van Rooij et al., 2018). Academic adaptation remains a context-specific construct involving academic cognitive, behavioral, and affective adjustment (Credé & Niehorster, 2012; Soares & Taveira, 2024).

Academic adaptation is finally expected to be positively associated with self-perceived adaptive intelligence. This association is theoretically plausible because students who perceive themselves as adjusting effectively to academic challenges may also perceive themselves as using flexible, creative, practical, and wisdom-related approaches in broader, uncertain situations. However, the cross-sectional model cannot determine whether academic adaptation precedes these broader self-perceptions or whether the relationship is reciprocal.

Accordingly, the two routes are retained as sequential mediation paths in the theoretical model specification, but their estimates represent theoretically ordered statistical indirect associations. They do not demonstrate that growth beliefs were sequentially converted into motivation or metacognition, then into self-regulation, academic adaptation, and adaptive functioning over time. Demonstrating such temporal unfolding would require longitudinal, multi-wave, or experimental evidence.

**H10a.**  *Academic growth mindset has a positive indirect association with self-perceived adaptive intelligence through learning motivation, self-regulated learning strategies, and academic adaptation.*

**H10b.**  *Academic growth mindset has a positive indirect association with self-perceived adaptive intelligence through metacognitive awareness, self-regulated learning strategies, and academic adaptation.*

#### 2.2.7. Learner Autonomy as a Moderator of the Self-Regulated Learning Strategies and Academic Adaptation Relationship

Learner autonomy is defined as students’ perceived ability to assume responsibility for their learning, make independent academic decisions, organize learning activities, and regulate progress (Macaskill & Taylor, 2010). Autonomy, self-regulation, decision-making, goal-setting, monitoring, and reflection are interrelated aspects of learner agency (Gupta et al., 2024). Students with higher autonomy may have greater discretion in setting goals, selecting resources, organizing study routines, seeking feedback, and adapting strategies (R. M. Ryan & Deci, 2020). On this basis, learner autonomy is hypothesized to strengthen the positive association between self-regulated learning strategies and academic adaptation. However, autonomy support may differ in its association with self-regulation depending on the amount of structure provided (Admiraal et al., 2024), making the moderation hypothesis empirically open rather than assumed.

**H11.**  *Learner autonomy positively moderates the association between self-regulated learning strategies and academic adaptation among students.*

## 3. Methods

### 3.1. Sampling Framework

The target population consisted of undergraduate students enrolled in universities in Jordan. According to the Ministry of Higher Education and Scientific Research (2026), Jordan has 28 universities, including 10 public and 18 private universities. All universities were initially contacted; however, access was obtained from nineteen universities, representing approximately 68% of the national university sector. The study, therefore, used a multi-institutional non-probability sample with purposive eligibility screening rather than probability sampling. Participation depended on institutional access and voluntary student participation; consequently, the resulting sample should not be treated as nationally representative of all Jordanian undergraduates.

Participating institutions included public and private universities located across the northern, central, and southern regions of Jordan. This coverage increased institutional heterogeneity but did not remove the limitations of convenience- and access-based recruitment. Students were eligible if they were enrolled in an undergraduate program at Level 2 or above, as they were expected to have sufficient experience with university learning requirements to respond meaningfully to the study variables. Generalization beyond the participating students and institutions should therefore be approached with caution.

The final sample comprised 640 undergraduate students. The sample exhibited a relatively balanced gender distribution, with 302 males (47.2%) and 338 females (52.8%). Most respondents were aged between 20 and 23 years (*n* = 472, 73.8%), followed by those under 20 years (*n* = 118, 18.4%) and those over 23 years (*n* = 50, 7.8%). The sample also showed variation in academic progression: Level 3 students constituted the largest group (*n* = 241, 37.7%), followed by Level 4 (*n* = 223, 34.8%) and Level 2 (*n* = 176, 27.5%). Regarding institutional context, 412 respondents (64.4%) were enrolled in public universities, while 228 (35.6%) were enrolled in private universities.

### 3.2. Data Collection Procedures

Data were collected using a self-administered questionnaire distributed across the participating Jordanian universities after obtaining the relevant approvals. The first page of the questionnaire explained the study purpose, assured respondents of confidentiality and anonymity, and clarified that participation was voluntary. Participants were also informed that their responses would be used only for academic research purposes. To minimize response bias, the questionnaire was organized into clear sections, instructions were provided before the measurement items, and respondents were asked to answer based on their actual university learning experiences. An initial screening question was included to confirm that respondents were enrolled at Level 2 or above.

The questionnaire was distributed in person by twenty trained research assistants through university networks and academic contacts. Course instructors and university contacts assisted only in facilitating access to eligible students and did not select participants on behalf of the researchers. A total of 678 questionnaires were returned. After screening for missing values, straight-lining, and unusable responses, 640 valid questionnaires were retained for analysis, representing a usable-response rate of 94.4% among returned questionnaires. Since the same questionnaire format was used across the participating universities and no separate online and paper subsamples were analyzed, no mode-comparison test was required. The final dataset was subsequently analyzed using WarpPLS 8.0. Prior step, ethical approval was obtained and participants provided informed consent before completing the questionnaire.

### 3.3. Measures

The survey items were adapted from well-established, previously validated instruments, with minor contextual modifications to align with the higher-education environment in Jordan. Growth mindset was evaluated using five items adapted from Lou and Noels (2017) and Collett and Berg (2020). Learning motivation was assessed using five items adapted from Vallerand et al. (1992). Metacognitive awareness was measured using six items adapted from the Metacognitive Awareness Inventory (Schraw & Dennison, 1994). Self-regulated learning strategies were measured using six items adapted from Pintrich et al. (1991, 1993). Learner autonomy was evaluated with five items drawn from the Autonomous Learning Scale developed for university students (Macaskill & Taylor, 2010).

Academic adaptation was conceptualized as a higher-order construct with three lower-order dimensions and nine items: cognitive adaptation, behavioral adaptation, and affective adaptation, adapted from Martin et al. (2012). The dependent variable was relabeled as self-perceived adaptive intelligence to more accurately reflect its measurement. It was modeled as a higher-order construct comprising 28 Likert-type items across wisdom-related adaptation, social/practical adaptation, creative reasoning, and uncertainty-navigation capacity. The item content was informed by Sternberg’s adaptive intelligence framework and the literature on adaptively intelligent attitudes (Sternberg, 2019, 2021; Sternberg et al., 2024). Accordingly, the scores represent respondents’ self-perceived adaptive tendencies and beliefs, not demonstrated performance or objectively measured intelligence. Negatively oriented items in the social/practical dimension were reverse-coded before analysis so that higher scores consistently indicated stronger self-perceived adaptive intelligence. All questionnaire items used a five-point scale from 1 (strongly disagree) to 5 (strongly agree).

### 3.4. Questionnaire Pretest

The validity of the questionnaire was established through a rigorous pretesting procedure designed to assess item clarity, readability, linguistic equivalence, and content relevance. Because the study targeted undergraduate students in Jordanian universities, special attention was given to ensuring that the wording of the items was understandable, contextually appropriate, and capable of eliciting accurate responses. Using the respondents’ native language was considered methodologically appropriate because it reduces cognitive burden, enhances response accuracy, and strengthens the reliability of self-reported data.

To ensure linguistic and conceptual equivalence, the questionnaire underwent a systematic translation and back-translation process. First, the original English version was translated into Arabic by a bilingual academic with expertise in educational research. Second, an independent bilingual specialist, who had no prior access to the original English version, translated the Arabic version back into English. All inconsistencies were discussed and resolved to ensure that the Arabic version preserved the original items’ semantic meaning while remaining culturally appropriate for Jordanian undergraduate students. This procedure followed established recommendations for cross-cultural instrument adaptation and back-translation (Brislin, 1970).

A six-member faculty panel reviewed the translated questionnaire for clarity, academic relevance, linguistic accuracy, and suitability for the study context. Their feedback led to minor refinements in terminology, sentence structure, and item wording to improve contextual alignment with the higher-education setting. These revisions helped ensure that the questionnaire items accurately reflected the constructs under investigation and were understandable to the target respondents. Additionally, Cross-language and content validity were also examined using the Content Validity Index (CVI). At the item level, the CVI values ranged from 0.87 to 0.91, indicating strong expert agreement regarding the relevance and clarity of individual items. The scale-level CVI was 0.921, which provides further evidence of excellent content validity. These results confirmed that the translated questionnaire achieved an acceptable level of expert consensus and was suitable for use in the main study.

Feedback from 70 undergraduate students in the same target population, collected during a pilot study, indicated that the questionnaire was generally clear and well-structured. Only minor clarifications to instructions and formatting were made; no substantive changes were made to the measurement items. Therefore, the pilot responses were retained in the final sample.

### 3.5. Common Method Bias

Because all study variables were collected through a single self-administered survey, common method bias was addressed through procedural controls and post hoc statistical analyses. Participants were assured of anonymity and confidentiality, informed that participation was voluntary, and told that there were no right or wrong answers. Clear instructions and logically structured sections were used to reduce ambiguity and respondent fatigue (Podsakoff et al., 2003). Nevertheless, the measures were predominantly self-reported and positively keyed, which may have increased consistency-related motifs and inflated positive inter-construct correlations; the statistical checks described below reduce, but do not eliminate, this concern.

Upon completion of data collection, statistical analyses were conducted to assess common method bias. Harman’s single-factor test, employing principal component analysis, demonstrated that the first unrotated factor accounted for only 18.04% of the total variance, significantly below the 50% threshold. Twelve factors exhibited eigenvalues exceeding 1, indicating that variance was distributed rather than concentrated in a single factor. These results provide preliminary evidence that common method bias did not pose a significant risk to the dataset. In addition to Harman’s test, collinearity diagnostics for all latent constructs within the structural model revealed variance inflation factor values below the conservative threshold of 5. This suggests that significant collinearity was absent, making it unlikely that the relationships among constructs were exaggerated due to common method variance (Hair et al., 2021).

Recognizing the limitations of Harman’s test, further variance-based evaluations were undertaken. Full collinearity assessment, an unmeasured latent method construct technique, and an alternative model containing a theoretically unrelated marker variable did not indicate substantial method-related contamination. These results suggest that common method variance was unlikely to account fully for the observed associations. They do not, however, establish that common method bias was absent, particularly because the constructs were measured at the same time, from the same respondents, and mainly with similarly keyed Likert items.

## 4. Results

### 4.1. Measurement Model Assessment

Partial least squares structural equation modeling (PLS-SEM) was used because the proposed model included multiple indirect paths, higher-order constructs, and a moderation term. WarpPLS 8.0 was employed with the PLS regression outer-model algorithm, the Warp3 inner-model algorithm, and the Stable3 standard-error estimation method. Stable3 is the software’s default and does not generate bootstrap resamples; therefore, no bootstrap sign-change handling was applied. The reported standard errors, t-ratios, *p*-values, and confidence intervals were based on Stable3 estimates. This clarification also explains why many indicator standard errors are similar (approximately 0.036–0.037).

For the higher-order constructs, a disjoint two-stage approach was applied. In the first stage, the lower-order dimensions were assessed for reliability and validity. In the second stage, their latent variable scores were used as indicators of the higher-order constructs—academic adaptation and self-perceived adaptive intelligence—before the structural associations were estimated.

The measurement model was evaluated by indicator reliability, internal consistency reliability, convergent validity, discriminant validity, and full collinearity. The results indicated adequate measurement quality across the constructs because all indicator loadings were statistically significant (*p* < 0.001), composite reliability values exceeded 0.70, and Cronbach’s alpha values exceeded the recommended threshold of 0.70 (Hair et al., 2021).

Table 1 reports reliability, convergent validity, and collinearity statistics. Composite reliability ranged from 0.861 to 0.905, and Cronbach’s alpha ranged from 0.758 to 0.878. The wisdom-related adaptation dimension had an AVE of 0.500, exactly at the conventional minimum threshold. It was retained because its composite reliability was strong (CR = 0.894), all indicator loadings were statistically significant, and the dimension has a central theoretical role in the higher-order construct. This borderline AVE should nevertheless be considered when interpreting the dimension. Full collinearity VIF values ranged from 1.066 to 2.347, below the conservative 3.3 criterion (Kock, 2025).

Indicator reliability was further assessed using the individual outer loadings shown in Table 2. The retained indicators loaded strongly on their intended constructs, with loadings ranging from 0.707 to 0.851 across the reflective constructs. All loadings were significant at *p* < 0.001, supporting indicator reliability and the adequacy of the item set for subsequent higher-order modeling.

Discriminant validity was assessed using the Fornell–Larcker criterion and HTMT ratios. As shown in Table 3, the square root of AVE for each lower-order construct exceeded its correlations with other constructs, supporting empirical distinctiveness.

As illustrated in Table 4, the HTMT results provided additional support for discriminant validity. All values were below 0.85, with the highest overall value being 0.619 between cognitive adaptation and self-regulated learning. Because conceptual overlap between academic adaptation and Self-perceived adaptive intelligence was a specific concern, cross-construct HTMT values were also examined between the three academic-adaptation dimensions and the four self-reported adaptive-functioning dimensions. These values ranged from 0.085 to 0.505; the highest was between cognitive adaptation and wisdom-related adaptation (0.505), well below the 0.85 criterion (Henseler et al., 2015). This pattern is consistent with the content distinction: academic-adaptation items refer specifically to cognitive, behavioral, and affective adjustment to university situations, whereas self-reported adaptive-functioning items assess broader perceived wisdom-related, social/practical, creative, and uncertainty-navigation tendencies. Appendix A provides further support for these results through the reported model fit and quality indices.

### 4.2. Structural Model Estimation

The structural model was assessed to examine the strength, direction, and statistical significance of the hypothesized associations among the study constructs. Standardized path coefficients and *p*-values were used for the direct associations, while R^2^ and adjusted R^2^ values were used to evaluate variance explained within the sample. Stone–Geisser’s Q^2^ values were used to assess predictive relevance. Effect sizes (f^2^) were interpreted using the conventional benchmarks of 0.02, 0.15, and 0.35 for small, medium, and large associations, respectively (Cohen, 1988).

The model demonstrated acceptable explanatory and predictive statistics for the endogenous constructs. Academic growth mindset accounted for 14.4% of the variance in learning motivation and 15.9% of the variance in metacognitive awareness. The predictors jointly accounted for 28.7% of the variance in self-regulated learning strategies and 42.2% of the variance in academic adaptation. Academic adaptation accounted for 27.5% of the variance in self-perceived adaptive intelligence. All Q^2^ values were above zero, ranging from 0.144 to 0.411, indicating predictive relevance within the analyzed sample (Hair et al., 2021).

#### 4.2.1. Direct Relationships

**Figure 2:**
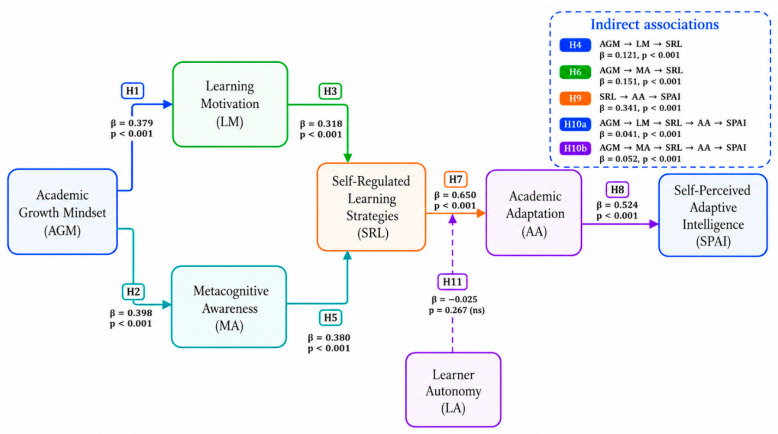
Results of the structural model. Source: Author’s preparation.

As shown in Table 5 and Figure 2, academic growth mindset was positively associated with learning motivation (β = 0.379, t = 9.974, *p* < 0.001, f^2^ = 0.144), supporting H1, and with metacognitive awareness (β = 0.398, t = 10.474, *p* < 0.001, f^2^ = 0.159), supporting H2. These coefficients indicate that stronger growth-mindset beliefs co-occurred with higher reported motivation and metacognitive awareness in the present sample.

Learning motivation was positively associated with self-regulated learning strategies (β = 0.318, t = 8.368, *p* < 0.001, f^2^ = 0.122), supporting H3. Metacognitive awareness was also positively associated with self-regulated learning strategies (β = 0.380, t = 10.000, *p* < 0.001, f^2^ = 0.166), supporting H5.

Self-regulated learning strategies were strongly associated with academic adaptation (β = 0.650, t = 17.568, *p* < 0.001, f^2^ = 0.417), supporting H7. Academic adaptation was positively associated with Self-perceived adaptive intelligence (β = 0.524, t = 14.162, *p* < 0.001, f^2^ = 0.275), supporting H8. These coefficients should be interpreted as contemporaneous structural associations, not as evidence that one construct caused a change in the next.

#### 4.2.2. Indirect Associations

##### Mediation Analysis

Indirect associations were evaluated in WarpPLS 8.0 using coefficients, Stable3-estimated standard errors, t-ratios, *p*-values, and 95% confidence intervals. Stable3 does not create bootstrap resamples; therefore, the confidence intervals reflect the Stable3 standard-error estimates rather than percentile bootstrap intervals. An indirect association was considered statistically significant when its confidence interval excluded zero. Although the hypothesized model specifies sequential mediation paths, the sequence follows theoretical ordering rather than repeated temporal observation (Hayes, 2018). Because the data were cross-sectional, statistical significance was interpreted as evidence of theoretically ordered indirect association, not temporal or causal mediation.

As shown in Table 6 and Figure 2, academic growth mindset was positively associated with learning motivation (β = 0.379, t = 9.989, *p* < 0.001, 95% CI [0.305, 0.453]), and learning motivation was positively associated with self-regulated learning strategies (β = 0.318, t = 8.312, *p* < 0.001, 95% CI [0.243, 0.392]). The indirect association through learning motivation was significant (β = 0.121, t = 6.411, *p* < 0.001, 95% CI [0.084, 0.158]), supporting H4. This result is interpreted as a statistically decomposed indirect association rather than evidence that learning motivation temporally mediates the relationship.

Academic growth mindset was positively associated with metacognitive awareness (β = 0.398, t = 10.520, *p* < 0.001, 95% CI [0.324, 0.473]), and metacognitive awareness was positively associated with self-regulated learning strategies (β = 0.380, t = 10.015, *p* < 0.001, 95% CI [0.306, 0.454]). The indirect association through metacognitive awareness was significant (β = 0.151, t = 7.233, *p* < 0.001, 95% CI [0.110, 0.192]), supporting H6.

#### Hypothesized Sequential Indirect Associations

Additional indirect associations were examined to further describe the structural model. Learning motivation was indirectly associated with academic adaptation through self-regulated learning strategies (β = 0.206, t = 7.630, *p* < 0.001, 95% CI [0.153, 0.259]). Metacognitive awareness was also indirectly associated with academic adaptation through self-regulated learning strategies (β = 0.247, t = 9.148, *p* < 0.001, 95% CI [0.194, 0.300]).

Academic adaptation was examined as an intervening variable in the association between self-regulated learning strategies and self-perceived adaptive intelligence. Self-regulated learning strategies were associated with academic adaptation (β = 0.650, t = 17.633, *p* < 0.001, 95% CI [0.578, 0.722]), and academic adaptation was associated with self-perceived adaptive intelligence (β = 0.524, t = 14.032, *p* < 0.001, 95% CI [0.451, 0.597]). The corresponding indirect association was significant (β = 0.341, t = 12.630, *p* < 0.001, 95% CI [0.288, 0.394]), supporting H9.

Academic growth mindset had a significant theoretically sequential indirect association with self-perceived adaptive intelligence through learning motivation, self-regulated learning strategies, and academic adaptation (β = 0.041, t = 5.542, *p* < 0.001, 95% CI [0.027, 0.056]). The corresponding sequential route through metacognitive awareness was also significant (β = 0.052, t = 6.048, *p* < 0.001, 95% CI [0.035, 0.069]), supporting H10a and H10b. The combined indirect association across the two routes was significant (β = 0.093, SE = 0.028, t = 3.321, *p* < 0.001, 95% CI [0.038, 0.148]). These results support the statistical coherence of the hypothesized sequential mediation structure; however, they do not demonstrate that the mediators operated in this temporal sequence.

#### 4.2.3. Moderation Analysis

**Figure 3:**
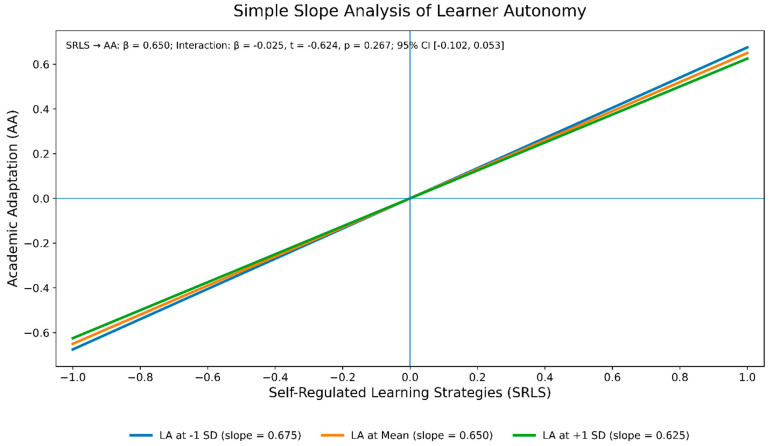
Interaction between learner autonomy and self-regulated learning strategies in relation to academic adaptation. Source: Authors’ preparation.

Learner autonomy was tested as a moderator of the association between self-regulated learning strategies and academic adaptation. The interaction coefficient was negative and statistically insignificant (β = −0.025, t = −0.624, *p* = 0.267, f^2^ = 0.005, 95% CI [−0.102, 0.053]). Because the confidence interval included zero, H11 was not supported, see Figure 3. Learner autonomy, therefore, did not significantly change the strength of the association in this sample.

The moderated indirect association was also statistically insignificant. Specifically, the indirect association of the interaction term with Self-perceived adaptive intelligence through academic adaptation was not significant (β = −0.013, t = −0.464, *p* = 0.322, f^2^ = 0.001, 95% CI [−0.068, 0.042]). Thus, learner autonomy did not condition the indirect association between self-regulated learning strategies and Self-perceived adaptive intelligence through academic adaptation in this sample.

## 5. Discussion

This study identified a coherent pattern of cross-sectional associations among academic growth mindset, learning motivation, metacognitive awareness, self-regulated learning strategies, academic adaptation, and self-perceived adaptive intelligence. Academic growth mindset was positively associated with both learning motivation and metacognitive awareness, consistent with mindset theory and prior evidence linking growth-oriented beliefs with students’ interpretations of effort, challenge, and feedback (Dweck, 2006; Dweck & Yeager, 2019). The results indicate that growth mindset covaried with both affective-motivational and cognitive-reflective learning resources; they do not show that mindset activated or produced those resources over time.

The positive associations of learning motivation and metacognitive awareness with self-regulated learning strategies indicate that reported strategic learning co-occurred with both willingness to engage and perceived capacity to monitor learning. Learning motivation reflects energy, persistence, and goal orientation, whereas metacognitive awareness reflects planning, evaluation, and strategy adjustment. The somewhat larger coefficient for metacognitive awareness suggests a stronger contemporaneous association with self-regulated learning strategies in this sample, but it should not be interpreted as evidence of causal priority.

Self-regulated learning strategies were strongly associated with academic adaptation. Students who reported planning, monitoring, time management, performance evaluation, and strategy adjustment also reported stronger cognitive, behavioral, and affective adjustment to academic demands. Academic adaptation is therefore better interpreted here as a domain-specific correlate and a statistical intervening variable, rather than a demonstrated developmental outcome of strategic learning behavior.

Academic adaptation was also positively associated with self-perceived adaptive intelligence. This finding is consistent with Sternberg’s view that adaptive intelligence includes context-sensitive problem solving, practical judgment, creativity, and wisdom-related orientations (Sternberg, 2021; Sternberg et al., 2024). However, the outcome in this study captured self-perceived tendencies rather than objectively measured ability. The association therefore indicates that students who perceived themselves as adjusting effectively to university demands also tended to describe themselves as more flexible, creative, practical, and attentive to consequences in uncertain situations. It does not show that academic adaptation developed an intellectual capacity.

The sequential indirect effect results likewise require an associational interpretation. Learning motivation and metacognitive awareness each accounted for significant indirect portions of the association between academic growth mindset and self-regulated learning strategies, and the longer theoretically ordered routes through self-regulated learning strategies and academic adaptation were also statistically significant. Thus, the sequential mediation model may be retained as a theory-based structural specification, but its support is limited to the covariance pattern observed in the single-wave data. The findings cannot establish temporal precedence, cumulative development, or causal mediation.

Learner autonomy did not significantly moderate the association between self-regulated learning strategies and academic adaptation. This finding suggests that autonomy may not serve as a boundary condition once reported self-regulation is taken into account, although alternative roles remain possible. Because self-regulated learning itself includes goal setting, planning, self-monitoring, and independent strategy adjustment, conceptual overlap may reduce the additional interaction contribution of learner autonomy. Autonomy may instead be associated with self-regulated learning directly or may operate as a contextual resource whose value depends on instructional structure (Admiraal et al., 2024; R. M. Ryan & Deci, 2020).

Overall, the results extend prior research by integrating beliefs, motivation, metacognition, self-regulation, academic adaptation, and broader self-perceived adaptivity into a single structural model. The evidence is specific to a non-probability sample of Jordanian undergraduates and should not be generalized to all Jordanian students or other national contexts without replication.

### 5.1. Implications

#### 5.1.1. Theoretical Implications

First, the findings extend mindset theory by showing that the academic growth mindset is embedded in a broader nomological network rather than operating solely as a direct correlate of student outcomes. Its associations with self-regulated learning strategies were statistically accounted for through both learning motivation and metacognitive awareness. This contribution concerns structural covariance and indirect associations; it does not establish that mindset initiates a developmental process.

Second, the study contributes to self-regulated learning theory by clarifying the complementary associations of motivation and metacognition with reported strategy use. Learning motivation reflects why students persist and invest effort, whereas metacognitive awareness reflects how they monitor and evaluate learning. The significant indirect associations through both variables support a multidimensional account of self-regulated learning.

Third, the study clarifies the role of academic adaptation as a domain-specific intervening variable between self-regulated learning strategies and self-perceived adaptive intelligence. Students’ adjustment to university demands was empirically distinguishable from broader wisdom-related, social/practical, creative, and uncertainty-navigation self-perceptions. This distinction avoids treating two adaptation-related constructs as interchangeable.

Fourth, the study contributes to adaptive intelligence research by explicitly distinguishing adaptive intelligence theory from its self-report operationalization. The measured outcome reflects perceived adaptive competencies or functioning, not cognitive performance. The results, therefore, demonstrate that these self-perceptions are associated with educational beliefs, strategies, and academic adjustment; claims about actual adaptive ability require performance-based assessment.

Fifth, the non-significant moderation by learner autonomy refines the proposed model. Autonomy did not change the strength of the association between self-regulated learning strategies and academic adaptation in this sample. Future theory may therefore consider autonomy as an antecedent, a direct correlate, or a feature of the learning environment rather than assuming that it necessarily functions as a moderator.

#### 5.1.2. Practical Implications

The findings also have practical implications, although the cross-sectional design does not establish that the proposed actions will cause changes in the study variables. Universities may nevertheless use the observed associations to inform programs that connect growth-oriented messages with strategy use, feedback seeking, reflection, and improvement. Instructors can frame mistakes as opportunities for learning and encourage students to revise ineffective approaches rather than interpret difficulty as evidence of fixed inability.

Academic courses may also provide structured opportunities for metacognitive practice, including reflective learning journals, guided self-questioning, exam wrappers, progress checklists, and post-assignment reflection. Such practices are theoretically aligned with the positive association between metacognitive awareness and self-regulated learning strategies. Academic support should additionally address digital distraction, which is associated with attention and self-regulation difficulties (Al-Abyadh et al., 2024).

Universities may integrate self-regulated learning skills into first-year programs, academic advising, and course design. Direct instruction in goal setting, time management, help seeking, resource use, and self-evaluation is consistent with the strong association observed between self-regulated learning strategies and academic adaptation.

Academic adaptation may be treated as an important student-support indicator. The results suggest value in services that help students manage academic uncertainty, assessment pressure, changing learning demands, and emotional challenges, including peer mentoring, academic coaching, early alert systems, transition workshops, and counseling focused on adjustment.

To strengthen capabilities relevant to adaptive intelligence theory, instructors may use problem-based learning, case studies, simulations, reflective projects, and real-world assignments that require the analysis of alternatives, the evaluation of consequences, and the revision of decisions. Future longitudinal or experimental research is needed to determine whether such practices improve performance-based adaptive ability or self-perceived adaptive intelligence.

Sixth, because learner autonomy did not significantly moderate the relationship between self-regulated learning strategies and academic adaptation, universities should avoid assuming that simply giving students more independence will improve adaptation. Autonomy should be structured through clear expectations, guided choices, constructive feedback, and scaffolded decision-making. Students benefit most when they are allowed to make learning decisions while still receiving adequate academic guidance and support.

For Jordanian higher education, the observed associations suggest that student-success initiatives may consider not only grades and retention but also motivation, metacognitive awareness, self-regulated learning, academic adjustment, and students’ perceived adaptive functioning. Because the sample was non-probabilistic, these implications should be treated as provisional and evaluated across additional institutions and student groups.

### 5.2. Limitations and Future Research

This study has several limitations. First, its single-wave, cross-sectional design does not establish temporal precedence or causality among academic growth mindset, learning motivation, metacognitive awareness, self-regulated learning strategies, academic adaptation, and self-perceived adaptive intelligence. Although the model retains theoretically specified sequential mediation paths, the term “sequential” refers only to the ordering imposed by the structural model. The indirect effects are statistical decompositions of contemporaneous associations and do not show that the mediators actually unfolded in the proposed order. Longitudinal cross-lagged, intensive longitudinal, multi-wave, or experimental designs are needed to examine temporal ordering, causal mediation, and developmental change.

Second, all focal constructs were measured through the same self-report questionnaire. Social desirability, consistency motifs, acquiescence, and the predominantly positive wording of items may have inflated inter-construct correlations. Although procedural remedies, full-collinearity diagnostics, a marker-variable model, and an unmeasured-method-factor analysis did not indicate severe contamination, these tests cannot rule out common method bias. Future studies should combine self-reports with academic records, teacher ratings, behavioral traces, learning analytics, interviews, and performance tasks.

Third, participants were recruited through purposive and convenience procedures from accessible universities in one country. Although the sample included institutional and regional variation, it was not nationally representative. Accordingly, the findings are sample-specific and should be generalized cautiously beyond the participating Jordanian undergraduates; replication is required across cultures, disciplines, institutions, and higher-education systems.

Fourth, learner autonomy was examined only as a moderator, but the insignificant moderation effect suggests that its role may be more complex. Future studies could test learner autonomy as an antecedent, mediator, or part of an autonomy-supportive learning climate.

Finally, the outcome was measured with Likert-type self-reports of wisdom-related, social/practical, creative, and uncertainty-navigation tendencies. It therefore represents Self-perceived adaptive intelligence rather than measured adaptive intelligence or demonstrated cognitive ability. Future research should combine these scales with performance-based tasks, scenario judgments, informant reports, and problem-solving simulations. Such work should also test the conceptual distinctiveness and predictive validity of academic adaptation and adaptive functioning over time.

## 6. Conclusions

This study tested an integrated structural model linking academic growth mindset with self-perceived adaptive intelligence through learning motivation, metacognitive awareness, self-regulated learning strategies, and academic adaptation among Jordanian undergraduates. The results identified significant direct and theoretically ordered sequential indirect associations, whereas learner autonomy did not moderate the association between self-regulated learning strategies and academic adaptation. The measured outcome reflects students’ self-perceived adaptive tendencies, informed by adaptive intelligence theory, rather than objectively assessed intellectual ability. Academic adaptation and self-perceived adaptive intelligence were conceptually and empirically distinguishable despite their positive association. This distinction was supported by cross-construct HTMT values ranging from 0.085 to 0.505, with the maximum value of 0.505 remaining well below the 0.85 criterion. The hypothesized sequential mediation structure is retained as a theory-based representation of the indirect paths; however, given the cross-sectional, single-source, non-probability design, the findings should be interpreted as sample-specific structural associations rather than evidence of temporal, causal, or developmental processes.

## Figures and Tables

**Table 1:** Construct reliability, convergent validity, and collinearity statistics.

Construct	Loading Range	CR	α	AVE	VIF	Decision
Academic growth mindset	0.739–0.781	0.872	0.816	0.576	1.475	Acceptable
Learning motivation	0.739–0.799	0.879	0.828	0.592	1.294	Acceptable
Metacognitive awareness	0.721–0.784	0.889	0.850	0.572	1.375	Acceptable
Self-regulated learning strategies	0.707–0.791	0.887	0.847	0.567	2.347	Acceptable
Learner autonomy	0.763–0.808	0.894	0.852	0.628	1.066	Acceptable
Cognitive adaptation	0.822–0.845	0.870	0.776	0.691	1.509	Acceptable
Behavioral adaptation	0.831–0.851	0.880	0.795	0.710	1.341	Acceptable
Affective adaptation	0.811–0.835	0.861	0.758	0.674	1.223	Acceptable
Wisdom-related adaptation	0.728–0.776	0.894	0.862	0.500	1.368	Acceptable
Social/practical adaptation	0.727–0.794	0.905	0.878	0.578	1.158	Acceptable
Creativity	0.719–0.774	0.895	0.863	0.549	1.313	Acceptable
Uncertainty navigation	0.761–0.776	0.879	0.828	0.592	1.140	Acceptable

Note: CR = composite reliability; AVE = average variance extracted; VIF = Variance Inflation Factor. Source: Authors’ preparation.

**Table 2:** Measurement loadings.

Code	Indicator	Loading	SE	*p*-Value
**Academic growth mindset**				
AGM1	I can always improve my ability to learn.	0.781	0.036	<0.001
AGM2	No matter how much learning ability I have, I can change it quite a bit.	0.741	0.037	<0.001
AGM3	I can become better at academic learning through practice and effort.	0.769	0.036	<0.001
AGM4	I believe that struggling with academic tasks helps me learn more effectively.	0.739	0.037	<0.001
AGM5	Making mistakes is part of improving my academic learning skills.	0.766	0.036	<0.001
**Learning motivation**				
LM1	Because I experience pleasure and satisfaction while learning new things.	0.770	0.036	<0.001
LM2	Because I find learning activities interesting.	0.772	0.036	<0.001
LM3	For the pleasure I experience when I discover new things.	0.739	0.037	<0.001
LM4	Because I like to learn new things.	0.799	0.036	<0.001
LM5	For the satisfaction I feel while learning.	0.766	0.036	<0.001
**Metacognitive awareness**				
MA1	I plan my learning before I begin.	0.770	0.036	<0.001
MA2	I monitor my understanding while learning.	0.784	0.036	<0.001
MA3	I know what strategies are most effective for me.	0.770	0.036	<0.001
MA4	I change strategies when I fail to understand.	0.721	0.037	<0.001
MA5	I evaluate how well I accomplish my learning goals.	0.741	0.037	<0.001
MA6	I am aware of my strengths and weaknesses when learning.	0.750	0.036	<0.001
**Self-regulated learning strategies**				
SRL1	I set goals for myself to direct my learning activities.	0.791	0.036	<0.001
SRL2	I ask myself questions to make sure I understand the material.	0.707	0.037	<0.001
SRL3	I try to change the way I study to fit the course requirements.	0.736	0.037	<0.001
SRL4	I make sure I keep up with the weekly readings and assignments.	0.753	0.036	<0.001
SRL5	I evaluate my progress toward my learning goals.	0.762	0.036	<0.001
SRL6	I manage my study time effectively.	0.764	0.036	<0.001
**Learner autonomy**				
LA1	I take responsibility for my own learning.	0.803	0.036	<0.001
LA2	I enjoy finding my own way of learning new things.	0.808	0.036	<0.001
LA3	I plan my learning activities independently.	0.763	0.036	<0.001
LA4	I actively seek opportunities to improve my learning.	0.798	0.036	<0.001
LA5	I am confident in my ability to manage my learning.	0.791	0.036	<0.001
**Academic adaptation**				
*Cognitive adaptation*				
CA1	I can consider several possible options when facing a new academic situation.	0.845	0.036	<0.001
CA2	I can revise my way of thinking to manage a new learning situation.	0.822	0.036	<0.001
CA3	I can adjust my expectations or thinking when academic conditions change.	0.826	0.036	<0.001
*Behavioral adaptation*				
BA1	I can seek new information, useful resources, or support to deal with new academic situations.	0.831	0.036	<0.001
BA2	In uncertain situations, I can develop new ways of completing academic tasks.	0.845	0.036	<0.001
BA3	I can change the way I do things when a new academic situation requires it.	0.851	0.036	<0.001
Affective adaptation				
AA1	I can reduce negative emotions, such as fear, when facing uncertainty.	0.811	0.036	<0.001
AA2	I can minimize frustration or irritation when academic uncertainty arises.	0.835	0.036	<0.001
AA3	I can use positive emotions, such as enjoyment or satisfaction, to help me manage new situations	0.816	0.036	<0.001
*Self-perceived adaptive intelligence*				
*Wisdom-related adaptation*				
WIS1	I consider other people’s feelings when making decisions.	0.750	0.036	<0.001
WIS2	When solving a problem, I consider how affected people may feel about my solution.	0.741	0.037	<0.001
WIS3	While solving a problem, I monitor whether my solution is working.	0.728	0.037	<0.001
WIS4	I trust my intuitive sense of right and wrong in almost any situation.	0.776	0.036	<0.001
WIS5	When I face problems, I find it useful to seek advice from others.	0.731	0.037	<0.001
WIS6	When thinking about solutions, I consider long-term consequences, not only short-term results.	0.728	0.037	<0.001
WIS7	I try to apply common sense in daily life, even when I feel tempted to do otherwise.	0.760	0.036	<0.001
WIS8	Before applying a solution, I check whether it is actually the right solution.	0.752	0.036	<0.001
WIS9	I focus on solutions that help me fit in with my peers.	0.727	0.039	<0.001
*Social/practical adaptation*				
SP1	When I have a problem, I often look for an authority figure to solve it for me. (RC)	0.756	0.036	<0.001
SP2	I do not mind being hypocritical because others are, too. (RC)	0.783	0.036	<0.001
SP3	I conform to social pressure because conformity helps people get ahead. (RC)	0.794	0.036	<0.001
SP4	People do best when they put their own interests first. (RC)	0.733	0.037	<0.001
SP5	I sometimes lie to others for their own sake so that I do not hurt their feelings. (RC)	0.779	0.036	<0.001
SP6	I sometimes ask whether the problem I am solving is the right problem.	0.747	0.036	<0.001
SP7	I prefer following clear instructions when solving problems.	0.727	0.037	<0.001
*Creativity*				
CR1	I enjoy finding new solutions to old problems.	0.774	0.036	<0.001
CR2	I often ask questions that nobody else asks.	0.727	0.037	<0.001
CR3	I am willing to try new ways of solving problems when old ways do not work.	0.768	0.036	<0.001
CR4	When I encounter an interesting new problem, I usually generate new ideas for it.	0.720	0.037	<0.001
CR5	Other people consider me someone who often has different or unusual ideas.	0.739	0.037	<0.001
CR6	I am generally open to new ideas and new ways of doing things proposed by others.	0.719	0.037	<0.001
CR7	I am willing to admit mistakes, even when doing so is embarrassing.	0.739	0.037	<0.001
*Uncertainty navigation*				
UN1	When facing an academic or life problem, I value perspectives from people who think differently from me.	0.772	0.036	<0.001
UN2	When I receive criticism, I consider whether it may help me improve my decision or solution	0.761	0.036	<0.001
UN3	I listen carefully to different viewpoints before deciding how to respond to a complex problem.	0.776	0.036	<0.001
UN4	I recognize that I may make mistakes when making decisions under uncertain conditions.	0.775	0.036	<0.001
UN5	When solving complex problems, I take enough time to evaluate possible consequences before acting.	0.765	0.036	<0.001

Source: Authors’ preparation.

**Table 3:** Fornell–Larcker matrix for constructs.

Construct	1	2	3	4	5	6	7	8	9	10	11	12
1. AGM	**0.759**											
2. MA	0.369	**0.770**										
3. LM	0.397	0.135	**0.756**									
4. SRL	0.439	0.367	0.423	**0.753**								
5. LA	0.200	0.108	0.052	0.144	**0.793**							
6. CA	0.284	0.208	0.304	0.502	0.096	**0.831**						
7. BA	0.211	0.311	0.171	0.453	−0.005	0.258	**0.842**					
8. AA	0.229	0.163	0.307	0.355	0.038	0.224	0.178	**0.821**				
9. WIS	0.200	0.238	0.194	0.460	0.121	0.408	0.219	0.155	**0.706**			
10. SP	0.152	0.159	0.125	0.316	0.091	0.220	0.262	0.107	0.208	**0.760**		
11. CR	0.220	0.163	0.189	0.429	0.055	0.392	0.213	0.148	0.278	0.228	**0.741**	
12. UN	0.188	0.102	0.164	0.290	0.057	0.165	0.083	0.252	0.088	0.051	0.168	**0.770**

Note: Values in bold are the square roots of AVE. Source: Authors’ preparation.

**Table 4:** HTMT ratios for constructs.

Construct	1	2	3	4	5	6	7	8	9	10	11	12
1. AGM												
2. MA	0.450											
3. LM	0.477	0.161										
4. SRL	0.528	0.437	0.499									
5. LA	0.241	0.127	0.085	0.170								
6. CA	0.357	0.260	0.375	0.619	0.118							
7. BA	0.262	0.383	0.208	0.551	0.051	0.328						
8. AA	0.291	0.206	0.383	0.442	0.048	0.292	0.230					
9. WIS	0.242	0.287	0.235	0.557	0.150	0.505	0.278	0.195				
10. SP	0.180	0.188	0.144	0.366	0.107	0.266	0.314	0.133	0.319			
11. CR	0.263	0.192	0.221	0.502	0.086	0.478	0.256	0.184	0.337	0.262		
12. UN	0.229	0.127	0.195	0.346	0.074	0.206	0.102	0.318	0.111	0.085	0.199	

Source: Authors’ preparation.

**Table 5:** Direct association testing results.

Hypo.	Structural Path	β	t-Value	*p*-Value	f^2^	Decision
H1	Academic growth mindset -> Learning motivation	0.379	9.974	<0.001	0.144	Supported
H2	Academic growth mindset -> Metacognitive awareness	0.398	10.474	<0.001	0.159	Supported
H3	Learning motivation -> Self-regulated strategies	0.318	8.368	<0.001	0.122	Supported
H5	Metacognitive awareness -> Self-regulated strategies	0.380	10.000	<0.001	0.166	Supported
H7	Self-regulated strategies -> Academic adaptation	0.650	17.568	<0.001	0.417	Supported
H8	Academic adaptation -> Self-perceived adaptive intelligence	0.524	14.162	<0.001	0.275	Supported

Source: Author’s preparation.

**Table 6:** Mediation results.

Hypo.	Indirect Path	β	t-Value	*p*	95% CI LL	95% CI UL	Decision
H4	Academic growth mindset -> Learning motivation -> Self-regulated strategies	0.121	6.411	<0.001	0.084	0.158	Supported
H6	Academic growth mindset -> Metacognitive awareness -> Self-regulated strategies	0.151	7.233	<0.001	0.110	0.192	Supported
H9	Self-regulated learning strategies -> Academic adaptation -> Self-perceived adaptive intelligence	0.341	12.630	<0.001	0.288	0.394	Supported
H10a	Academic growth mindset -> Learning motivation -> Self-regulated learning strategies -> Academic adaptation -> Self-perceived adaptive intelligence	0.041	5.542	<0.001	0.027	0.056	Supported
H10b	Academic growth mindset -> Metacognitive awareness -> Self-regulated learning strategies -> Academic adaptation -> Self-perceived adaptive intelligence	0.052	6.048	<0.001	0.035	0.069	Supported
Combined 4-segment	Academic growth mindset -> Learning motivation/Metacognitive awareness -> Self-regulated learning strategies -> Academic adaptation -> Self-perceived adaptive intelligence	0.093	3.321	<0.001	0.038	0.148	Supported

Source: Authors’ preparation.

## Data Availability

The data supporting the findings of this study are available from the corresponding author upon reasonable request, subject to ethical and privacy restrictions.

## Appendix A

**Table A1:** Model Fit and Quality Indices.

Index	Value	*p*-Value	Interpretation
APC	0.382	<0.001	Acceptable
ARS	0.257	<0.001	Acceptable
AARS	0.256	<0.001	Acceptable
AVIF	1.089	-	Acceptable (<5; ideally <= 3.3)
AFVIF	1.508	-	Acceptable (<5; ideally <= 3.3)
Tenenhaus GoF	0.393	-	Large
SPR	1.000	-	Acceptable
RSCR	1.000	-	Acceptable
SSR	1.000	-	Acceptable
NLBCDR	1.000	-	Acceptable

Source: authors’ preparation.
